# Personal, behavioural and socio-environmental correlates of emerging adults’ sustainable food consumption in a cross-sectional analysis

**DOI:** 10.1017/S1368980023000654

**Published:** 2023-06

**Authors:** Elizabeth Ludwig-Borycz, Dianne Neumark-Sztainer, Nicole Larson, Ana Baylin, Andrew D Jones, Allison Webster, Katherine W Bauer

**Affiliations:** 1 Department of Nutritional Sciences, University of Michigan, Ann Arbor, MI 48109, USA; 2 Division of Epidemiology and Community Health, University of Minnesota, Minneapolis, MN, USA; 3 Department of Epidemiology, University of Michigan, Ann Arbor, MI, USA; 4 International Food Information Council Foundation, Washington, DC, USA

**Keywords:** Sustainable food consumption, Planetary Health Diet, Nutrition, Dietary intake, Young adult, Plant-based food, Animal-sourced food

## Abstract

**Objective:**

Describe how dietary intake patterns of US young adults align with the EAT-Lancet Planetary Health Diet (PHD) sustainable diet goals and identify personal, behavioural, and socio-environmental correlates of sustainable intake.

**Design:**

Data on past-year dietary intake were captured using a FFQ. The PHD was applied to specific food groups, and a total PHD score was calculated. Linear regression models were used to identify associations between personal, behavioural and socio-environmental factors and PHD scores.

**Setting:**

This cross-sectional analysis uses data from the second wave of EAT 2010–2018 (Eating and Activity over Time), a population-based longitudinal study recruited in Minnesota.

**Participants:**

Ethnically/racially diverse group of participants (*n* 1308) with a mean age of 22·1 (sd 2·0) years.

**Results:**

The mean PHD score was 4·1 (sd 1·4) on a scale of 0–14, with 14 representing the most sustainable. On average, participants consumed fewer whole grains, fish, legumes, soya, and nuts than ideal for a sustainable diet, and an excess of eggs, added sugar, and meat. The PHD score was higher for participants with higher socio-economic status (SES) and greater educational attainment. Higher home availability of healthy food (*β* = 0·24, *P* < 0·001) and less frequent fast-food consumption (*β* = –0·26, *P* < 0·001) were the strongest correlates of PHD scores.

**Conclusions:**

Results suggest that a high percentage of participants may not be achieving the sustainable diet goals defined by the PHD. Reductions in meat consumption and increases in plant-based foods are necessary to increase the sustainability of US young adults’ diets.

In 2015, the Paris Agreement set the goal to limit the global temperature increase to less than 2°C to mitigate the devastating effects of climate change^(1)^. Sustainable food systems are essential to meeting the goal of the Paris Agreement because agriculture is responsible for about 25 % of greenhouse gas emission globally, more than 70 % of freshwater use^(2)^, 80 % of deforestation^(3)^, and is the single largest contributor to biodiversity loss^(4)^. A sustainable food system involves diets that provide for both the flourishing of human and environmental health and are affordable, equitable, safe, and culturally appropriate^(5)^. Individuals can support sustainable food systems by consuming a diet comprised of foods that arise from sustainable practices.

As the prevalence of overweight and obesity has increased beyond 2 billion globally^(6)^, another 2 billion individuals remain micronutrient-deficient^(7)^ and 821 million individuals are undernourished (habitual insufficient energetic intake)^(8)^. Identifying ways to optimise human health that fit within safe planetary boundaries is imperative both to combat climate change and meet nutritional needs^(9)^. Globally, nations are working to mitigate climate change and maximise human nutrition by incorporating sustainability into their dietary recommendations. The EAT-Lancet Commission brought together experts in the fields of human health, agriculture, political science and environmental sustainability to help meet the Sustainable Development Goals and Paris Agreement, which allows for feeding an estimated 10 billion people globally by 2050^(2)^. The EAT-Lancet Planetary Health Diet (PHD)^(2)^ was developed by the EAT-Lancet Commission in 2019 as one approach to establish an adaptable metric with which to assess diet sustainability in a manner that simultaneously recognises the environmental and health impacts of consumption of various food groups. The PHD was designed to be healthy for humans and the environment with regard to greenhouse gas emission, nitrogen and phosphorus application, agricultural water use, biodiversity loss, and cropland use^(10)^ and relies predominantly on plant-based foods which is consistent with a recent literature review^(11)^.

In 2019, Wang et al. estimated that 25 % of premature deaths could be prevented if the US populations consumed diets that aligned with the PHD^(12)^. Notwithstanding, some shortcomings of the diet have been noted, particularly in relation to the affordability of the PHD. Calculated as costing an average of US$2·65 per d in 2011, the PHD is affordable for the vast majority of US population groups^(13)^ even so, many Americans may find the PHD challenging to adopt as it differs from current US dietary patterns^(14)^ and affordability does not necessarily translate to accessibility.

However, few studies have assessed the extent to which national dietary recommendations and current intake patterns of US populations align with the PHD goals. This assessment is important as the Dietary Guidelines for Americans (DGA) are used to inform many federal nutrition programmes and public health strategies targeting health promotion and disease prevention. Blackstone and Conrad^(15)^ identified that the 2015–2020 DGA fall below recommendations for sustainable dietary intake based on the PHD, and a recent analysis of US school lunches served at elementary, middle and high schools found that these meals were particularly low in whole grains and vegetables, while high in meat and dairy products, when compared with the PHD^(16)^. These findings suggest that US nutrition programmes and actual dietary intake may likely be substandard with respect to diet sustainability, particularly when measured by the PHD.

Further, the factors that support consumption of sustainable diets have not been rigorously examined. A small number of large population-based studies conducted among adults in the USA, France and Poland have identified that individuals who consume more sustainable diets have a lower BMI^(17)^, engage in more physical activity^(18)^, consume less fast food and alcohol^(17,19)^, and overall, have better diet quality^(17,19,20)^. Additionally, studies among adults in Denmark and Belgium show that sustainable diets are more common among higher socio-economic status (SES) groups including those with higher educational attainment, higher income and food security^(21,22)^. None of these studies used the PHD as a measure of diet sustainability, highlighting the need for a standardised measure to assess sustainable dietary intake.

The objective of the current study is to assess diet sustainability among a large, racially/ethnically diverse population-based sample of young adults recruited from a large metropolitan area of Minnesota by comparing their dietary intake to the targets of the PHD. Young adults hold particular importance since they are at a life stage of increasing independence and are developing habits that may persist throughout their adult lives^(23)^. Additionally, we identify personal, behavioural and socio-environmental correlates of young adults’ sustainable dietary intake assessed via the PHD. One study found that less than 35 % of young adults value sustainable diet practices and that greater value for sustainable diet practices was associated with higher diet quality, greater intake of vegetables and less fast food consumption^(19)^. Therefore, we hypothesise that most young adult participants have substandard sustainable dietary intake based on the PHD and that sustainable dietary intake will correlate with other health-promoting behaviours (e.g. physical activity, sleep and low fast-food consumption). The knowledge obtained from this study will provide the first benchmark regarding sustainable dietary intake using the PHD among a young adult US sample from Minnesota and suggest intervention targets that could reduce barriers to and promote sustainable food consumption across diverse communities.

## Methods

### Study population

The current cross-sectional analysis uses data from the second wave of EAT 2010–2018 (Eating and Activity over Time), a population-based study designed to understand weight-related health across the life course. EAT 2010 was conducted within the Minneapolis and St. Paul school districts of Minnesota, USA^(24)^. Consideration was given to involvement in other research studies and enrolling an ethnically/racially diverse sample of adolescents when identifying schools for participation in the EAT 2010 study. Two urban school districts, which served a large number of schools and diverse students, were invited to participate and twenty schools within these districts were recruited after the study was approved by the school district research boards. Survey dates were scheduled with teachers at each school, and EAT staff visited school classrooms at least 10 d prior to survey administration in order to distribute parent consent forms. Adolescents in health, physical education and science classes were given the opportunity to assent just prior to survey administration only if their parent/guardian did not return a signed consent form indicating their refusal to have their child participate. Among adolescents who were at school on the days of survey administration, 96·3 % had parental consent and chose to participate. The enrolled student sample (*n* 2793) was similar in terms of ethnic/racial composition to the overall student population within each district in 2010 based on data maintained by the Minnesota Department of Education. Students received a $10 Target gift card as compensation for their participation in the study. The mean age of participants was 14·4 years (sd = 2·0)^(24)^. In 2017–2018, a follow-up study was conducted, and EAT 2010 participants were invited to complete another survey and FFQ. There were 2383 EAT 2010 participants that were invited to take part in the study (410 were lost to follow-up) and 1568 responded by completing a survey online or by mail^(24)^. To account for missing data due to attrition, inverse probability weighting was used^(25)^. The current analysis included only the participants who completed both the survey and FFQ, excluding those who reported biologically implausible energetic intake (consuming < 400 or > 7000 kcal/d) (*n* 175). Participants with missing values for covariates (age, gender, income, education, race and total energetic intake) were also excluded to ensure comparability among models, resulting in a final sample of 1308 young adults; see Supplementary Figure 1 for a flow diagram of the analytic sample. The sample was more diverse than the overall population in Minneapolis–St. Paul, Minnesota with 20·8 % White, 20·6 % Asian American, 17·1 % Hispanic, 26·5 % African American or Black and 11·5 % mixed or other.

### Assessment of personal, behavioural and socio-environmental variables

The EAT surveys were developed to integrate an ecological perspective with Social Cognitive Theory. Personal, behavioural and socio-environmental variables (see Table 1) for this analysis were identified based on Social Cognitive Theory and on our existing understanding of predictors of sustainable diet intake within each of the Social Cognitive Theory domains^(33)^. Understanding the personal, behavioural and socio-environmental correlates of the PHD would identify subgroups of individuals that are consuming more sustainable diets and could suggest policy-based, environmental, and educational levers with the potential to move other groups towards more sustainable intake. To promote ease of interpretation, all variables were standardised to a mean of 0 and sd of 1.

**Table 1:** Assessment of personal, behavioural and socio-environmental factors

Variables	Definition
Personal	
BMI	Self-reported weight and height (kg/m^2^)^(24)^
Cooking self-efficacy	Including asking about people’s confidence doing five activities: planning meals, following a recipe, preparing a meal from items on hand, using basic cooking techniques and staying within a food budget, with a range of 5–25^(26)^
Depressive symptoms	Including feeling too tired to do things; having trouble going to sleep or staying asleep; feeling unhappy, sad or depressed; feeling hopeless about the future; feeling nervous or tense; worrying too much about things, with a range of 6–18^(24)^
Unmanaged stress	The average level of stress in the past month divided by ability to manage stress in the past month with a range of 0·1–10^(27)^
Self-esteem	Six items from the Rosenberg Self-esteem Scale, including *I am satisfied with myself; I have a number of good qualities; at times I think that I am no good at all; able to do most things as well as most other people; wish I could have more respect for myself; and I certainly feel useless at times* with a range of 10–24^(24)^
Body satisfaction	Satisfaction with your height, weight, body shape, waist, hips, thighs, stomach, face, body build, shoulders, muscles, chest and overall body fat with a range of 13–65^(28)^
Behavioral	
Mindful eating	Including eating so quickly that I don’t taste what I’m eating; snacking without noticing that I am eating; taking a moment to appreciate the colours and smells of my food; tasting every bite of food that I eat with a range of 4–16^(29)^
Fast-food intake	Number of times you ate fast food (including burger, Mexican, fried chicken, pizza and Asian) over the past month with a range of 0–140^(24)^
Eating at a restaurant	Number of times you ate at a restaurant (including all fast-food plus sit-down restaurants) over the past month with a range 0–168^(24)^
Physical activity	Hours per week engaging in moderate to vigorous activity, ranging from 0 to 16^(24)^
Alcohol consumption	Derived from the FFQ in grams per d
Screen time	Average hours of recreational screen time (e.g. television, computer, social media, video games, smartphone or tablet) per week with a range of 7–42 accounting for weekdays and weekends^(24)^
Sleep hours	Average hours per d derived from asking when do you usually go to bed and get out of bed^(24)^
Lifestyle weight management behaviours	Number of lifestyle weight management behaviours performed last year, including exercise, eating fruits and vegetables, eating less high-fat foods, eating less sweets, drinking less soda pop, drinking more water, watching portion sizes, and other^(30)^
Unhealthy weight control behaviours	Number of unhealthy weight control behaviours performed last year including fasted, eating very little food, taking diet pills, vomiting, using laxatives, taking diuretics, using food substitutes, skipping meals and smoking cigarettes^(30)^
Socio-environmental	
Home healthy food availability	Three items were used to assess whether the following were available at home (‘Please think about the apartment, house, dorm room, or other space where you lived for the majority of the time for the past year’): fruits and vegetables were available, vegetables are part of the dinner meal and whole wheat bread is available with a range of 3–12.^(24)^ Response options were *Never, Sometimes, Usually* and *Always.*
Parental encouragement of healthy eating	Mother(father) encourages me to eat healthy foods with a range of 4–16^(31)^
Support for healthy eating and PA at work	Five items were used to assess whether participants could easily be physically active at or around their workplace, co-workers think it is important to be physically active, co-workers care about eating healthy food, easy to buy healthy food at or around the workplace, and employees rarely bring high-calorie foods with a range of 5–20^(32)^
Household food security	Two items from the US Household Food Security Survey Module: 1) ‘In the past 12 months did you ever eat less than you felt you should because there wasn’t enough money for food?’ and 2) ‘In the past 12 months were you ever hungry but didn’t eat because there was not enough money for food?’. Response options were *yes, no* and *I don’t know*. If the participant said yes to both household food security questions, they were determined to be food-insecure^(24)^

### Assessment of diet

A semi-quantitative 149-item validated FFQ was administered at the same time as the EAT survey to assess usual dietary intake in the past year^(34)^. To compare intake to the PHD criteria, the scoring method developed by Hanley-Cook et al.^(35)^ with minimum intake values was applied. Participants’ intake was categorised into one of the fourteen PHD food groups (Supplemental Table 1), and conversion factors reported by Blackstone et al.^(15)^ were used to translate from servings per d to grams per d (1 serving fruit = 182 g; 1 serving dark green vegetables = 118 g; 1 serving red and orange vegetables = 114 g; 1 serving starchy vegetables = 134 g; 1 serving other vegetables = 140 g; 1 serving whole grains = 51 g; 1 serving dairy products = 149 g; 1 serving meat = 31 g; 1 serving poultry = 29 g, 1 serving eggs = 50 g; 1 serving fish = 29 g; 1 serving nuts and seeds = 15 g; 1 serving soya = 24 g, and 1 serving legumes = 44 g). In accordance with Hanley-Cook et al.^(35)^, a score of 1 was given for each food group when average daily intake fell within the following ranges: whole grains (232·0–464·0 g/d), tubers (50·0–100·0 g/d), dairy products (250·0–500·0 g/d), beef, lamb and pork (14·0–28·0 g/d), chicken and other poultry (29·0–58·0 g/d), eggs (13·0–25·0 g/d), fish (28·0–100·0 g/d), dry beans, lentils, peas (50·0–100·0 g/d), soya (25·0–50·0 g/d), peanuts or tree nuts (25·0–100·0 g/d), added fat (20·0–91·8 g/d), and added sugar (0·0–31·0 g/d). A score of 0 was given to those who were outside (both below and above) the PHD intake range^(35)^. An exception was made for vegetables and fruits, which only had a minimum intake without a maximum intake in accordance with Knuppel et al.^(36)^ so as to not penalise high consumption of fruits and vegetables. For vegetables and fruits, a score of 1 was given to those who met or exceeded the minimum intake (≥ 200 g/d) and (≥ 100/d), respectively, while a score of 0 was given to those who fell short of the PHD^(36)^.

The PHD was developed to align with daily energy intake of 2500 kcal/d. To standardise the application of the PHD to the total energetic intake of participants, their intake in grams was scaled to 2500 kcal/d. In contrast to this method, a sensitivity analysis was conducted by weighting the PHD to align with a 1500 kcal/d intake and 2000 kcal/d intake creating ideal intake goals for three ranges: < 1500 kcal/d, 1500–2500 kcal/d and > 2500 kcal/d. The results of the sensitivity analysis (online Supplementary Tables 2–4) were similar to the analysis based on energy intake of 2500 kcal/d when participants’ individual intake in grams was scaled based on energy intake, demonstrating the robustness of the findings.

### PHD score

The primary outcome, overall PHD score, was created in accordance with Hanley-Cook et al.^(36)^ by summing points for achieving optimal intake in each of fourteen food categories derived from the FFQ, resulting in an index with possible scores ranging from 0 to 14, with 0 being the least sustainable and 14 being the most sustainable. Furthermore, percent difference of participant intake from the PHD for each of the food categories was calculated by subtracting the midpoint of the suggested PHD energetic range from the observed participant intake weighted by that participant’s ideal intake range^(2)^.

### Sociodemographic characteristics

Ethnicity/race was determined by asking ‘Do you think of yourself as White, Black or African American, Hispanic or Latino, Asian American, American Indian or Native American, or Other’. Socio-economic status was classified using participants’ highest level of parental education along with eligibility for public assistance, free or reduced-price school lunches, and parental employment status. Gender, educational attainment, birth year and student status were self-reported^(24)^.

### Statistical analysis

Descriptive statistics were used to examine PHD scores (overall and for each food group) across participant characteristics, including age, gender, ethnicity/race, educational attainment, SES, student status and total energy intake. The authors calculated means and standard deviations of PHD scores, the percent of participants achieving the PHD goals, percent below the PHD goal and percent exceeding the PHD goal. The differences in mean PHD composite score across sociodemographic groups (gender, ethnicity/race, educational attainment and SES) were compared using ANOVA. Linear regression models were then constructed to allow for separately examining each personal, behavioural and socio-environmental factor of interest as a predictor of PHD composite score. Model assumptions were checked prior to running the models. Crude models were first constructed and then further adjusted for potential confounders in alignment with previous studies, including ethnicity/race, educational attainment, gender, age, SES and total energy intake^(19,21,22)^. A *P*-value of < 0·05 was used to indicate statistical significance. Statistical analyses were carried out in SAS version 9.4.

## Results

The weighted descriptive characteristics of the study sample in 2018 are presented in Table 2. The mean age of study participants was 22·1 (sd = 2·0), and just under half (41·8 %) were enrolled in college. Over half of participants (59·8 %) were of low- or low-middle SES.

**Table 2:** Sociodemographic characteristics of Project EAT 2018 participants (*n* 1349)

	%	Mean	sd
Age (years)		22·1	2·0
Gender			
Male	46·2		
Female	53·2		
Other	0·6		
Ethnicity/race			
White	20·8		
Black or African American	26·5		
Hispanic or Latino	17·1		
Asian American	20·6		
American Indian or Native American	3·6		
Mixed or other	11·5		
Educational attainment			
Some high school	5·3		
High school graduate or GED	29·2		
Some college	39·3		
Associate degree, vocational, technical or trade	11·4		
Bachelor’s, graduate or professional degree	14·8		
Socio-economic status			
Low	37·4		
Low-middle	22·4		
Middle	18·2		
Upper-middle	13·8		
High	8·3		
Student status			
Not a student	55·0		
Student in high school	3·2		
Student at a community or technical college	18·9		
Student at a 4-year college	20·7		
Graduate student	2·2		
Total energetic intake			
≤ 1500 kcal/d	37·0		
1500–2500 kcal/d	32·3		
≥ 2500 kcal/d	30·8		

Participants’ overall PHD score was 4·1 on average (sd = 1·4), on a scale of 0 to 14 possible, with 14 being the most sustainable (Table 3). Participants of low socio-economic status had significantly lower overall PHD scores (4·1 (sd = 1·4)) than those of high SES (4·5 (sd = 1·2)). Likewise, those with lower educational attainment, only some high school education, had lower overall PHD scores (3·9 (sd = 1·5)) than those with greater educational attainment, an associate, vocational, technical, trade, bachelor’s, graduate or professional degree (4·3 (sd = 1·4)).

**Table 3:** Planetary Health Diet scores by sociodemographic characteristics

	Planetary Health Diet score		F-statistic	*P*-value
	Mean	sd		
Gender			2·63	0·07
Male	4·1	1·5^a^		
Female	4·2	1·3^a^		
Other	4·2	1·0^a^		
Ethnicity/race			3·02	0·01
White	4·3	1·2^a^		
Black or African American	4·0	1·7^a^		
Hispanic or Latino	4·3	1·4^a^		
Asian American	4·0	1·2^a^		
American Indian or Native American	4·2	1·4^a^		
Mixed or other	4·3	1·4^a^		
Educational attainment			3·51	0·007
Some high school	3·9	1·5^ab^		
High school graduate or GED	4·0	1·4^a^		
Some college	4·2	1·4^b^		
Associate degree, vocational, technical or trade	4·3	1·4^ab^		
Bachelor’s, graduate or professional degree	4·3	1·4^ab^		
Socio-economic status			2·72	0·03
Low	4·1	1·4^a^		
Low-middle	4·1	1·4^ab^		
Middle	4·1	1·5^ab^		
Upper-middle	4·2	1·1^ab^		
High	4·5	1·2^b^		

Note: Means with common superscript letters do not differ at *P* < 0·05.

**Fig. 1:**
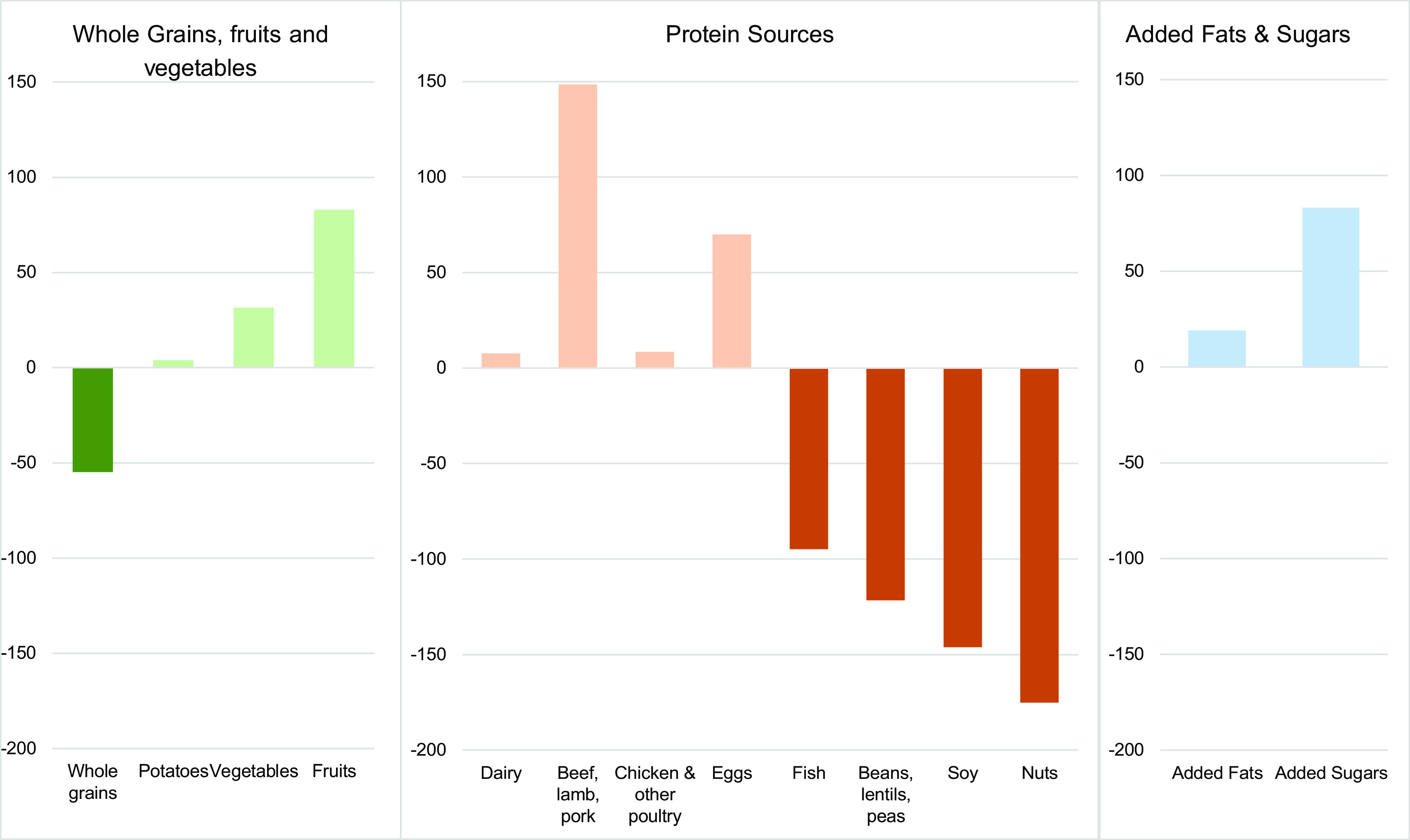
Difference of Project EAT 2018 Participant Intake from Planetary Health Diet Targets

Figure 1 shows the percent difference between the average intake of participants for each food group compared with the ideal PHD intake. Overall, participants were close to meeting PHD recommendations for potatoes (3·9 %), dairy products (7·7 %) and poultry (8·6 %). However, on average, participants over-consumed meat (148·5 %), eggs (70·0 %), and added sugar (83·2 %), and under-consumed whole grains (–54·8 %), fish (–94·7 %), legumes (–121·5 %), soya (–146·0 %) and nuts (–175·2 %). The mean scaled intake of meat is high at 47·4 (sd = 32·6) g/d with more than 71 % of participants consuming above the PHD recommendations. In comparison, the mean scaled intake of fish was 10·0 (sd = 12·8) g/d, and mean scaled intakes of plant-based proteins were 12·2 (sd = 20·4) g/d for legumes, 3·9 (sd = 11·9) g/d for soya and 3·3 (sd = 7·2) g/d for nuts, with more than 90 % of participants having intakes that were below PHD recommendations across all four categories (Table 4).

**Table 4:** Planetary Health Diet for Project EAT 2018 Participants

Dietary component	Planetary Health Diet Intake Goals in grams/d	Observed intake in g/d		Intake in g/d scaled to 2500 kcal/d		% Achieving PHD	% Below PHD	% Above PHD
		Mean	sd	Mean	sd			
Whole grains	232·0–464·0	115·6	124·2	132·2	112·0	10·5	87·7	1·8
Potatoes	50·0–100·0	46·6	64·9	52·0	50·9	28·5	59·1	12·4
Vegetables	≥ 200·0	359·9	447·4	412·3	375·0	69·7	30·3	N/A
Fruits	≥ 100·0	433·4	612·4	483·4	448·9	89·8	10·2	N/A
Dairy products	250·0–500·0	238·3	277·6	270·1	229·1	28·8	58·6	12·6
Beef, lamb and pork	14·0–28·0	41·1	43·8	47·4	32·6	17·8	10·5	71·7
Chicken and other poultry	29·0–58·0	26·4	31·6	31·6	27·9	27·8	58·9	13·3
Eggs	13·0–25·0	23·4	38·0	27·0	34·8	21·1	42·9	36·0
Fish	28·0–100·0	9·7	18·5	10·0	12·8	8·6	91·4	0·0
Beans, lentils and peas	50·0–100·0	10·9	22·8	12·2	20·4	3·8	95·3	0·9
Soya	25·0–50·0	3·2	9·3	3·9	11·9	2·8	96·3	0·9
Nuts	25·0–100·0	3·1	10·7	3·3	7·2	1·2	98·6	0·2
Added fats								
Added fat	20·0–91·8	55·2	39·4	62·7	18·2	94·3	0·9	4·8
Added sugars								
Added sweetener	≤ 31·0	66·8	65·3	75·2	48·3	12·2	N/A	87·8

Note: As min intake ranges were used in this analysis in alignment with Hanley-cook et al.^(29)^, neither % below nor % above the PHD is considered ideal.

Participants’ overall adjusted PHD scores were most strongly associated with standardised (mean = 0, sd = 1) scores indicating higher availability of healthy food at home (*β* = 0·24, *P* value < 0·001) and less frequent fast-food consumption (*β* = –0·26, *P* value < 0·001) (Table 5). Other personal characteristics associated with the PHD score were greater self-efficacy for cooking (*β* = 0·16, *P* value < 0·001), self-esteem (*β* = 0·10, *P* value = 0·009) and overall body satisfaction (*β* = 0·12, *P* value = 0·008). Increased hours of physical activity per week (*β* = 0·15, *P* value = 0·0002) and number of lifestyle weight management behaviours performed last year (*β* = 0·11, *P* value < 0·0001) were behavioural characteristics associated with more sustainable dietary intake. Meanwhile, less frequently eating at a restaurant (*β* = –0·25, *P* value < 0·0001) and fewer hours of screen time (*β* = –0·16, *P* value < 0·0001) were associated with sustainable dietary intake. Finally, participants reporting greater parental encouragement of healthy eating (*β* = 0·15, *P* value = 0·0002) experienced higher overall PHD scores on average, while participants experiencing food insecurity had moderately lower PHD scores (*β* = –0·09, *P* value = 0·02). The remaining personal (BMI, depressive symptoms and unmanaged stress), behavioural (mindful eating, alcohol consumption, hours of sleep per d and number of unhealthy weight control behaviours performed last year) and socio-environmental characteristics (support for healthy eating and physical activity at work) were not associated with the PHD score.

**Table 5:** Associations between personal, behavioural and socio-environmental characteristics* and Planetary Health Diet score†

Characteristics	*β*	se	*P*-value
Personal			
BMI (kg/m^2^)	0·01	0·04	0·70
Cooking skills	0·16	0·04	< 0·0001
Depressive symptoms	−0·05	0·04	0·20
Unmanaged stress	−0·07	0·04	0·08
Self-esteem	0·10	0·04	0·009
Overall body satisfaction	0·12	0·04	0·008
Behavioural			
Mindful eating	0·06	0·04	0·15
Monthly frequency of fast-food consumption	−0·26	0·04	< 0·0001
Monthly frequency of eating at a restaurant	−0·25	0·04	< 0·0001
Hours of physical activity per week	0·15	0·04	0·0002
Alcohol consumption grams per day	−0·02	0·04	0·56
Hours of screen time per week	−0·16	0·04	< 0·0001
Hours of sleep per d	−0·05	0·04	0·20
Number of lifestyle weight management behaviours performed last year	0·11	0·02	< 0·0001
Number of unhealthy weight control behaviours performed last year	0·02	0·03	0·34
Socio-environmental			
Home healthy food availability	0·24	0·04	< 0·0001
Parental encouragement of healthy eating	0·15	0·04	0·0002
Support for healthy eating and physical activity at work	0·05	0·05	0·28
Food-insecure	−0·09	0·04	0·02

* Personal, behavioural and socio-environmental predictors have been standardised to mean = 0, sd = 1 to allow for comparison of estimates across models.

† Models adjusted for ethnicity/race, educational attainment, gender, age, socio-economic status (SES) and total energy intake.

## Discussion

The objective of the current study was to assess intake of a sustainable dietary pattern among a large, socio-economically and ethnically/racially diverse sample of US young adults by comparing it to the targets of the PHD. Additionally, we identified personal, behavioural and socio-environmental correlates of young adults’ sustainable dietary intake assessed via the PHD. Overall, as hypothesised, young adults participating in EAT 2018 were not consuming diets that aligned with PHD recommendations. While most young adults met the PHD recommended intakes for fruits, vegetables and added fats, the majority under-consumed whole grains, plant-based proteins, and fish, and overconsumed meat and added sugar. Young adults of high SES and those with higher educational attainment consumed diets more aligned with PHD recommendations than their peers. Furthermore, the strongest correlates of meeting the PHD recommendations were greater healthy food availability at home and less frequently consuming food from fast-food restaurants.

Study findings are consistent with dietary patterns observed in other high-income countries (HIC) and contrast with patterns observed in low-to-middle-income countries with regard to meat and whole-grain consumption. For example, prior research using the cross-sectional nationally representative National School Lunch Program data found that the average amount of food prepared for by elementary, middle and high school cafeterias exceeded the PHD for dairy products, fruit, refined grains, red meat, and starchy vegetables and was insufficient for whole grains, legumes, vegetables and nuts^(16)^. An additional study in the UK has shown relatively few individuals meet the PHD recommendations for whole grains (36·1 %) and most met (66·6 %) or exceeded (33·4 %) the recommendations for meat^(36)^. In India, consumption expenditures for urban and rural populations, respectively, show that the PHD recommendations were exceeded for whole grains 1029 kcal/d and 1275 kcal/d and fell short of meeting recommendations for meat 3 kcal/d and 5 kcal/d, fish 8 kcal/d and 9 kcal/d, and eggs 6 kcal/d and 10 kcal/d^(37)^. A primary difference between the study conducted in India and the studies in the USA and UK are the discrepancies in animal-source food consumption and whole grains. In the USA and UK, the PHD recommendations are widely met or exceeded for animal-sourced foods, while in India they fall short of meeting them. Conversely, in India, the PHD recommendation is exceeded for whole grains, while in the USA and UK they fall short of meeting it^(36,37)^. These patterns mirror common dietary patterns among low-to-middle-income countries and HIC globally, which necessitates a shift in consumption in order to meet sustainability goals^(38)^. In low-to-middle-income countries, meeting the dual planetary and human health sustainability goals requires a higher intake of animal-based protein to replace some of the energy content they are getting from whole grains (especially to meet the nutritional needs of women and children in low-to-middle-income countries)^(35)^, while HIC need to reduce meat consumption and supplement it with a greater intake of whole grains and plant-based protein.

In HIC like the USA, reducing meat consumption and increasing intake of plant-based sources of protein provide a clear path for making gains in the sustainability of dietary intake. Such a change would likely also be economically advantageous for consumers, although not all scholars agree, and exceptions can be found. A 2021 Global Modelling Study found that in HIC vegetarian and vegan diets were on average more affordable than current dietary patterns by up to 34 %^(39)^. In the current study, young adults with the lowest SES consumed the most meat (beef, lamb and pork) in comparison with higher SES groups. This pattern is often observed within HIC^(40)^. One reason that individuals from lower SES households may consume more meat, and thus have lower overall PHD scores, is more frequent fast-food consumption (e.g. burgers). Among young adults in the EAT 2010–2018 study, fast-food consumption was one of the strongest correlates of lower diet sustainability. A recent study demonstrated the positive association between income and processed meat consumption; furthermore, it showed an additive interaction between income, neighbourhood density of fast-food outlets and the outcome of interest, processed meat consumption^(41)^. One innovative intervention strategy to improve the sustainability of low SES individuals’ diets is encouraging fast-food restaurants to showcase plant-based proteins, particularly ones that keep costs low. In 2021, seven fast-food restaurants (Burger King, Chipotle, Starbucks, KFC, Panera Bread, Pizza Hut and Taco Bell) were recognised for leading the way in plant-based protein alternatives in alignment with their corporate commitments to reducing meat consumption^(42)^. However, proximity to fast food is only one structural barrier that may contribute to the increased meat consumption among those in lower SES households; other potential structural barriers are food access, time constraints, perceived cost, cooking knowledge, taste and cultural preferences. Poole et al.^(16)^ examined the perceived cost barrier and found that school lunches meeting the PHD recommendations in the USA were less expensive than those that did not. Another study in Baltimore City examined taste as a barrier and found that a shift to eating PHD meals was well accepted by low-income families on the basis of taste, appearance and healthfulness of meals^(43)^.

Beyond shifts towards plant-based protein in the fast-food industry, fiscal policies known to alter the healthfulness of diets would likely also positively impact consumers’ PHD score^(44)^. For example, during the COVID-19 pandemic, the USA increased benefits for the Program for Women, Infants, and Children (WIC) from $9 per child and $11 per adult to $35 per person, and an evaluation found that participating children increased their fruit and vegetable intake after the benefit bump occurred^(45)^. Continuing this programme’s expanded benefits into the future may help improve the accessibility of healthful and sustainable diets to low-income families in the USA. Additionally, the USA could adopt other fiscal policies such as a sugar-sweetened beverages tax. The WHO recommends at least a 20 % tax on sugar-sweetened beverages and other unhealthy foods to be coupled with comparable subsidies on nutrient-dense foods like fruit, vegetables, whole grains, legumes and nuts as a method to shift consumption patterns, especially among low-income groups^(46)^. A case study can be found in Mexico, back in 2013 the government levied a 10 % sugar-sweetened beverages tax that reduced consumption by almost 10 %^(47)^. In contrast to this approach, the USA currently subsidises commodity crops that are frequently used to produce unhealthy foods many of which are a source of added sugar.

Another important component to help people in the USA consume more sustainable diets is ensuring that the DGA consider the shared goals of improving physical and environmental health. This is particularly important as a growing number of people are turning to the DGA for nutritional advice^(48)^, and the current DGA have similar or poorer environmental sustainability compared with current US dietary intake^(11)^. Notably, the 2015–2020 Dietary Guidelines Advisory Committee recommended that sustainability be considered as part of the DGA, but this recommendation was removed from the final guidelines as it was deemed beyond the scope of the Committee’s charge^(49)^. The most recent iteration of the DGA, 2020–2025, did not revisit the topic, and currently, the DGA allow for a much higher consumption of meat, refined grains and discretionary energy content than does the PHD^(15)^. The DGA also inform many federal nutrition programmes that supplement the diets of low SES individuals. As our study found that lower-income people had lower diet sustainability, bringing the DGA closer in alignment with the PHD could bolster the diet sustainability of lower SES individuals.

While this study had multiple strengths including a large population-based sample in Minnesota and socio-economically and racially/ethnically diverse participants, an important limitation was the brief assessment of plant-based proteins on the FFQ. This may have led to an underestimation of participants’ soya intake, resulting in lower overall PHD scores. Future research focused on assessing sustainable diets should ensure that their measures of dietary intake more comprehensively capture plant-based protein consumption. Participants were drawn from only one area in the USA, thereby limiting the generalisability of study findings to other young adult populations outside of the Minneapolis/St. Paul area of Minnesota. Additionally, as this study was cross-sectional, causality cannot be determined. Participants may have also over-reported behaviours or characteristics they perceived as socially acceptable and under-reported behaviours or characteristics they perceived as socially unacceptable due to social desirability. This would have the effect of attenuating the correlations of personal, behavioural and socio-environmental characteristics with the PHD.

The majority of young adults participating in the EAT 2010–2018 study had substandard sustainable dietary intake based on the PHD. This was particularly true for individuals of lower SES and educational attainment. Most young adults consumed high amounts of meat, a dietary behaviour that is especially harmful to the environment. Reducing meat consumption, especially by substituting plant-based proteins, is an important target for intervention among US young adults. Policy and environmental changes known to improve diet healthfulness such as taxing sugar-sweetened beverage and other unhealthy foods, subsidising nutrient-dense foods, fast-food restaurants committing to reducing meat consumption, and including sustainability into the DGA hold promising potential for shifting diets towards more environmentally sustainable choices.

## Supplementary material

For supplementary material accompanying this paper visit https://doi.org/10.1017/S1368980023000654. click here to view supplementary material
