# Supplementary material for: Personal, behavioural and socio-environmental correlates of emerging adults’ sustainable food consumption in a cross-sectional analysis
Source: Public Health Nutr. 2023 Apr 4;26(6):1306–16. doi: 10.1017/S1368980023000654 (PMC10346036; doi:10.1017/S1368980023000654)
Supplement: Supplementary file 1 [file S1368980023000654sup001.docx]

**Supplemental Table 1: Planetary Health Diet**

| **Dietary Component** | | **Food Item based on the FFQ** |
| --- | --- | --- |
|  |  |  |
| 1 | Whole Grains | Brown rice, whole wheat, brown bread, etc. |
| 2 | Potatoes and Cassava | Potatoes and french fries |
| 3 | Vegetables | Broccoli, kale, mustard greens, chard, spinach, romaine lettuce, leaf lettuce, bok choy, tomatoes, carrots, yams or sweet potatoes, dark orange winter squash, string beans, cauliflower, cabbage or coleslaw, brussels sprouts, corn, mixed or stir-fry vegetables, eggplant, zucchini or other squash, iceberg or head lettuce, celery, peppers, onions, mushrooms, radish, jicama, hot peppers, asparagus, beets, kimchee, cucumber, bamboo shoots, seaweed, okra, scallions, peapods |
| 4 | Fruits | Raisins or grapes, prunes or dried plums, prune juice, bananas, cantaloupe, fresh apples or pears, apple juice or cider, oranges, orange juice, grapefruit or grapefruit juice, other fruit juices, strawberries, blueberries, peaches or plums, apricots, cherries, melon, applesauce, papaya, mango, rhubarb, mixed dried fruit, figs, dates, fruit cocktail, pineapple, pomegranate, rhubarb, watermelon, dried cranberries, plantain, kiwi, raspberries, dried apple |
| 5 | Dairy Foods | Milk, yogurt, cheese, etc. |
| 6 | Beef, Lamb, and Pork | Hamburger, pork, hotdog, bacon, bologna, etc. |
| 7 | Chicken and other poultry | Chicken and turkey |
| 8 | Eggs | Eggs |
| 9 | Fish | Tuna, dark fish, shrimp, lobster, scallops, other fish |
| 10 | Dry beans, lentils, and peas | Beans, peas, hummus |
| 11 | Soy | Tofu, soy milk |
| 12 | Tree Nuts & Seeds | All nuts and seeds from FFQ |
| 13 | Added Fat | Entire FFQ totals |
| 14 | Added Sweeteners | Entire FFQ totals |

**Supplemental Table 2. Sensitivity Analysis Using Intake Categories: Planetary Health Diet for Project EAT 2018 Participants**

| **Dietary component** | **Planetary Health Diet Intake Goals in Grams/Day by Energy Intake** | | | **Observed**  **Intake in**  **g/day**  **(Mean (SD))** | **% Achieving PHD** | **% Below PHD** | **% Above PHD** |
| --- | --- | --- | --- | --- | --- | --- | --- |
|  | **<1,500 kcal/day** | **1,500-2,500 kcal/day** | **>2,500 kcal/day** |  |  |  |  |
| Whole grains | 139.0-278.0 | 186.0-371.0 | 232.0-464.0 | 115.6 (124.2) | 12.3 | 85.3 | 2.4 |
| Potatoes | 30.0-60.0 | 40.0-80.0 | 50.0-100.0 | 46.6 (64.9) | 15.6 | 68.9 | 15.5 |
| Vegetables | >120.0 | >160.0 | >200.0 | 359.9 (447.4) | 65.5 | 34.5 | N/A |
| Fruits | >60.0 | >80.0 | >100.0 | 433.4 (612.4) | 87.7 | 12.4 | N/A |
| Dairy | 150.0-300.0 | 200.0-400.0 | 250.0-500.0 | 238.3 (277.6) | 24.2 | 60.8 | 15.0 |
| Beef, lamb, pork | 8.0-17.0 | 11.0-22.0 | 14.0-28.0 | 41.1 (43.8) | 22.5 | 11.6 | 65.9 |
| Chicken & other poultry | 17.0-35.0 | 23.0-46.0 | 29.0-58.0 | 26.4 (31.6) | 25.2 | 61.3 | 13.5 |
| Eggs | 8.0-15.0 | 10.0-20.0 | 13.0-25.0 | 23.4 (38.0) | 15.6 | 46.4 | 38.0 |
| Fish | 17.0-60.0 | 22.0-80.0 | 28.0-100.0 | 9.7 (18.5) | 8.3 | 90.7 | 1.0 |
| Beans, lentils, peas | 30.0-60.0 | 40.0-80.0 | 50.0-100.0 | 10.9 (22.8) | 3.3 | 95.1 | 1.6 |
| Soy | 15.0-30.0 | 20.0-40.0 | 25.0-50.0 | 3.2 (9.3) | 3.0 | 96.3 | 0.7 |
| Nuts | 15.0-60.0 | 20.0-80.0 | 25.0-100.0 | 3.1 (10.7) | 1.5 | 98.3 | 0.2 |
| **Added Fats** |  |  |  |  |  |  |  |
| Added fat | 12.0-55.1 | 16.0-73.4 | 20.0-91.8 | 55.2 (39.4) | 79.6 | 3.7 | 16.7 |
| **Added Sugars** |  |  |  |  |  |  |  |
| Added sweetener | <19.0 | <25.0 | <31.0 | 66.8 (65.3) | 18.7 | N/A | 81.3 |

**Supplemental Table 3. Sensitivity Analysis Using Intake Categories: Planetary Health Diet Scores by Sociodemographic Characteristics**

|  | **Planetary Health Diet Score (Mean (SD))** | **F-statistic (P-Value)** |
| --- | --- | --- |
| **Gender** |  | 3.34 (0.04) |
| Male | 3.7 (1.5)^a^ |  |
| Female | 3.9 (1.3)^b^ |  |
| Other | 4.0 (1.5)^ab^ |  |
| **Ethnicity/race** |  | 2.90 (0.01) |
| White | 3.8 (1.2)^ab^ |  |
| Black or African American | 3.7 (1.7)^a^ |  |
| Hispanic or Latino | 4.0 (1.5)^ab^ |  |
| Asian American | 3.7 (1.3)^ab^ |  |
| American Indian or Native American | 3.6 (1.3)^ab^ |  |
| Mixed or other | 4.1 (1.3)^b^ |  |
| **Educational Attainment** |  | 2.12 (0.08) |
| Some high school | 3.6 (1.5)^a^ |  |
| High school graduate or GED | 3.7 (1.5)^a^ |  |
| Some college | 3.8 (1.3)^a^ |  |
| Associate degree, vocational, technical, or trade | 4.0 (1.3)^a^ |  |
| Bachelor’s, graduate, or professional degree | 3.9 (1.3)^a^ |  |
| **Socioeconomic Status** |  | 2.99 (0.02) |
| Low | 3.7 (1.4)^a^ |  |
| Low-middle | 3.9 (1.4)^ab^ |  |
| Middle | 3.8 (1.4)^ab^ |  |
| Upper-middle | 3.9 (1.3)^ab^ |  |
| High | 4.1 (1.2)^b^ |  |

Note: Means with common superscript letters do not differ at p<.05.

**Supplemental Table 4. Sensitivity Analysis Using Intake Categories: Associations between Personal, Behavioral, and Socio-environmental** **Characteristics^1^ and Planetary Health Diet Score**

| **Characteristics** | $\boldsymbol{\beta}$ **(SE)** | **P-value** |
| --- | --- | --- |
| **Personal** |  |  |
| BMI (kg/m^2^) | -0.007 (0.04) | 0.85 |
| Cooking skills | 0.20 (0.04) | <0.001 |
| Depressive symptoms | -0.06 (0.04) | 0.10 |
| Unmanaged stress | -0.08 (0.04) | 0.03 |
| Self-esteem | 0.12 (0.04) | 0.002 |
| Overall body satisfaction | 0.06 (0.04) | 0.13 |
| **Behavioral** |  |  |
| Mindful eating | 0.12 (0.04) | 0.001 |
| Monthly frequency of fast-food consumption | -0.22 (0.04) | <0.001 |
| Monthly frequency of eating at a restaurant | -0.20 (0.04) | <0.001 |
| Hours of physical activity per week | 0.19 (0.04) | <0.001 |
| Alcohol consumption grams per day | -0.009 (0.04) | 0.81 |
| Hours of screen time per week | -0.15 (0.04) | <0.001 |
| Hours of sleep per day | -0.03 (0.04) | 0.37 |
| Number of lifestyle weight management behaviors performed last year | 0.11 (0.02) | <0.001 |
| Number of unhealthy weight control behaviors performed last year | 0.03 (0.03) | 0.23 |
| **Socio-environmental** |  |  |
| Home healthy food availability | 0.26 (0.04) | <0.001 |
| Parental encouragement of healthy eating | 0.19 (0.04) | <0.001 |
| Support for healthy eating and physical activity at work | 0.07 (0.04) | 0.12 |
| Food Insecure | -0.08 (0.04) | 0.03 |

^1^Personal, behavioral, and socio-environmental predictors have been standardized to mean = 0, SD = 1 to allow for comparison of estimates across models.

^2^Models adjusted for ethnicity/race, educational attainment, gender, age, SES, and total energy intake

**Supplemental Figure 1: Sample Size Flow Chart**

Participants with biologically implausible caloric intake were excluded (**n=175).** Eating less than 400 or more than 7,000 kcal/day. Sample size that was used in the descriptive analysis (table 1 and 2) **N=1,349**

Participants who completed both the FFQ and Survey in 2018 **n=1,568**

Participants with missing values for covariates SES (29), education (8), and race (4) in the regression analysis were excluded (n=41) to ensure comparability among models (**n=1,308).**

Furthermore, exposures of interest with missing values were excluded in their respective models.

Personal Variables: Cooking skills **(n=1,302)**; Stress & stress management **(n=1,283)**; BMI **(n=1,286)**; Depression **(n=1,306)**; Self-esteem **(n=1,306)**; Overall body image **(n=1,296)**

Behavioral Variables: Mindful eating **(n=1,299)**; Fast-food consumption **(n=1,299)**; Eating at a restaurant **(n=1,300)**; Physical activity **(n=1,306)**; Screen time **(n=1,300)**; average sleep per day **(n=1,231)**; lifestyle weight management **(n=1,295)**; unhealthy weight control **(n=1,286)**

Social Variables: Healthy home food availability **(n=1304)**; Parental encouragement of healthy eating **(n=1,292)**; Food Insecurity **(n=1,293)**; Support for health eating and physical activity at work **(n=899)**
